# Computed tomography evaluation of alterations in the masticator space due to invasion by malignant head and neck neoplasms

**DOI:** 10.1590/0100-3984.2023.0024-en

**Published:** 2023

**Authors:** Mariana Luiza Bittencourt Campinhos, Otavio Alberto Curioni, Aldemir Humberto Soares, Marcelo Marcucci

**Affiliations:** 1 Faculdade de Odontologia da Universidade de São Paulo (FOUSP), São Paulo, SP, Brazil; 2 Hospital Heliópolis, São Paulo, SP, Brazil

**Keywords:** Tomography, X-ray computed, Neoplasms, Masticatory muscles, Neoplasms, Trismus., Tomografia computadorizada, Tumor maligno, Espaço mastigador, Trismo.

## Abstract

**Objective:**

To evaluate alterations in the masticator space due to the dissemination of malignant neoplasms originating from the tonsillar fossa, retromolar trigone, maxillary sinus, or nasopharynx, using computed tomography (CT), as well as to correlate the presence of trismus with the CT findings and the dimensions of the tumor.

**Materials and Methods:**

We evaluated the medical records of 65 patients with malignant tumors in the regions described. The images were analyzed by two physician examiners, working independently, who were blinded to the clinical data. In the evaluation of the masticator space, the following parameters were considered: symmetry with the contralateral space; obliteration of the fat plane, retromolar trigone, or pharyngeal space; edema/atrophy of the medial or lateral pterygoid muscles; and destruction of the mandibular ramus.

**Results:**

Obliteration of the fat plane was found in 69.2% of the patients. Asymmetry, edema/atrophy, and bone destruction were detected in 27.7%, 26.2%, and 20.0% of the patients, respectively. Trismus was identified in 15.4% of the patients. Of the patients with trismus, 90.0% had stage T4 tumors, compared with only 43.8% of those without trismus. Trismus was 11.6 times more common among the patients with stage T4 tumors than among those with lower-stage tumors. Neoplasms of the tonsillar fossa and retromolar trigone collectively accounted for 95.0% of the cases. The CT scans showed edema/atrophy of the pterygoid muscles in 60.0% of the patients with trismus and in 21.8% of those without. An association was observed between T4 tumor stage and edema/atrophy of the pterygoid muscles. In addition, the risk of trismus was 5.4 times higher among the patients with stage T4 tumors.

**Conclusion:**

In our patient sample, the most common finding was obliteration of the fat plane, followed by asymmetry and edema/atrophy. Most of the patients with T4 tumors had trismus, together with edema/atrophy of the pterygoid muscles.

## Abstract (pt)

**Objetivo:**

Avaliar, por meio de tomografia computadorizada, alterações do espaço mastigador (EM) decorrentes de disseminação de neoplasias malignas originárias da loja tonsilar, trígono retromolar, seio maxilar e nasofaringe, e correlacionar presença de trismo com achados tomográficos e dimensões do tumor.

**Materiais e Métodos:**

Foram selecionados prontuários de 65 pacientes portadores de tumores malignos nas regiões descritas. A análise das imagens foi realizada por dois examinadores médicos, separadamente, sem o conhecimento das informações clínicas. Na avaliação do EM, foram considerados: simetria com o EM contralateral; obliteração do plano gorduroso, do trígono retromolar e do espaço faríngeo; edema e/ou atrofia dos músculos pterigóideos medial e lateral; destruição do ramo da mandíbula.

**Resultados:**

Foram observados obliteração do plano gorduroso em 69,2% dos pacientes, assimetria em 27,7%, espessamento/atrofia em 26,2% e destruição óssea em 20,0%. Presença de trismo foi encontrada em 15,4% dos pacientes. Na associação entre dimensão do tumor e trismo, foram observados trismo em 90,0% dos tumores em estágio T4, enquanto a porcentagem de tumores em estágio T4 sem trismo foi de 43,8%. Pacientes com tumores T4 apresentaram 11,6 vezes mais trismo que os dos demais estágios. Neoplasias da loja tonsilar e trígono retromolar perfizeram 95,0% dos casos. Em 60,0% dos pacientes com trismo havia edema e/ou atrofia dos músculos pterigóideos na tomografia computadorizada e em 21,8% nos sem trismo. Observou-se associação entre tumores T4 e edema e/ou atrofia dos músculos pterigóideos e 5,4 vezes mais chance de apresentarem trismo.

**Conclusão:**

A maioria dos pacientes apresentou obliteração do plano gorduroso, seguido de assimetria e espessamento/atrofia. O trismo estava presente na maioria dos pacientes T4 com espessamento/atrofia dos músculos pterigóideos.

## INTRODUCTION

The masticatory space is a deep fascial space with a complex anatomical structure. It is divided into two compartments-medial and lateral-and encompasses the four masticatory muscles: the masseter, medial pterygoid, lateral pterygoid, and temporalis muscles. The space also encompasses bone tissue, including the mandibular ramus and the posterior portion of the mandibular body.

Some head and neck neoplasms, mainly those for which the primary site is the retromolar trigone, tonsillar fossa, nasopharynx, or maxillary sinus, can evolve to affect the masticator space through locoregional invasion. Understanding the anatomy of this space and how it communicates with other deep spaces helps radiologists identify the spread of tumors that affect this region. Because the masticator space is difficult to explore through physical examination, it is necessary to use imaging tests, such as magnetic resonance imaging (MRI) and computed tomography (CT). CT is a well-established diagnostic imaging method for assessing the extent of tumors and the involvement of adjacent structures^**(^1^)**^. In the masticator space, certain alterations, such as asymmetry in relation to the contralateral masticator space, obliteration of the fat plane, edema/atrophy of the masticatory muscles, and destruction of the mandibular ramus, are indicative of the presence of a malignant tumor and can be identified on CT or MRI scans^**(^2^)**^.

Restricted mouth opening has occasionally been seen in patients with head and neck cancer. Primary carcinomas are more likely to cause trismus than are metastases and benign neoplasms^**(^3^)**^. The presence of trismus in patients is associated with tumor involvement of the masticatory muscles^**(^4^-^8^)**^, which can make intraoral physical examination difficult. Given that primary tumors in this region are rare, the development of trismus is typically due to extension of the tumor to the masticator space, which induces muscle spasm^**(^9^)**^.

Given the importance of the combination of trismus and malignant head and neck neoplasms, the objectives of this study were to evaluate, by means of CT, alterations in the structures of the masticator space, due to locoregional dissemination of malignant neoplasms originating in the tonsillar region, retromolar trigone, sinus maxilla, or nasopharynx, and to correlate the CT findings with the presence of trismus and with the dimensions of the primary tumor.

## MATERIALS AND METHODS

### Subjects

This was a retrospective study based on the medical records of selected patients with malignant neoplasia in the region of the retromolar trigone, tonsillar fossa, maxillary sinus, or nasopharynx, diagnosed between July 2010 and October 2012, obtained from the files of the Department of Head and Neck Surgery of the Hospital Heliópolis, a public hospital operated by the Brazilian Unified Health Care System in the city of São Paulo, Brazil. The study was approved by the Research Ethics Committee of the University of São Paulo.

The inclusion criteria were being ≥ 18 years of age, having a diagnosis of malignant neoplasm confirmed by pathological examination, and having a CT examination available for interpretation. Patients who had previously undergone antineoplastic therapy (chemotherapy, radiotherapy, surgery, or any combination of those) were excluded, as were those with local inflammatory or infectious disease.

All of the medical records were reviewed by the same examiner, who collected information regarding age, gender, location of the primary tumor, time since disease onset, tumor-node-metastasis stage (based on the 2010 edition of the Union for International Cancer Control staging system), and the presence or absence of trismus. Trismus was defined as a patient complaint of restricted mouth opening, confirmed through inspection by a medical professional at the time of the initial physical examination.

### Image selection

The CT scans of the selected patients were retrieved from the digital archives of the Radiology Department of the Hospital Heliópolis. All CT scans were acquired in a single-slice helical scanner (Somatom Emotion; Siemens, Erlangen, Germany). After intravenous administration of iodinated contrast medium (Henetix 300; Guerbet, Villepinte, France), the following acquisition protocol was applied: 3-mm thick axial slices, acquired in parallel planes, perpendicular to the trachea; slices in the coronal plane acquired when necessary; acquisition time, 60 s; matrix, 512 × 512; tube voltage, 130 kVp; tube current, 90 mAs; and field of view, 258 mm. The images obtained were stored in a Digital Imaging and Communications in Medicine system (National Electrical Manufacturers Association, Rosslyn, VA, USA).

### Image analysis

The images of the masticator space were analyzed by means of scanning, at a workstation, the entirety of the axial sections being evaluated, as well as the entirety of any coronal sections that had been acquired. The evaluation was carried out by two examiners who were resident physicians in the third year of their radiology residency. The two examiners worked independently, and both were blinded to the clinical data. The findings were transcribed into a specific spreadsheet. According to criteria established by Wei et al.^**(^5^)**^, the presence or absence of the following items was noted: symmetry with the contralateral masticator space; obliteration of the fat plane, retromolar trigone, or pharyngeal space; edema/atrophy of the medial or lateral pterygoid muscles; and destruction of the mandibular ramus.

### Statistical analysis

Initially, the data were analyzed descriptively. Categorical variables are expressed as absolute and relative frequencies. For numerical variables, summary measures (mean, quartiles, minimum, maximum, and standard deviation) were calculated; the minimum, maximum, and quartile values are presented in a box plot. Because of the small sample size, associations between two categorical variables were quantified by using Fisher’s exact test. Comparisons of the means between two groups were made by using the nonparametric Mann-Whitney test.

Agreement between CT findings was determined by calculating the kappa statistic (κ). To evaluate the strength of the agreement, the following criteria were used^**(^9^)**^: ≤ 0.0 = poor; 0.00-0.20 = weak; 0.21-0.40 = fair; 0.41-0.60 = moderate; 0.61-0.80 = excellent; 0.81-1.00 = almost perfect.

For all statistical tests, a significance level of 5% was adopted. Statistical analyses were performed with the SPSS Statistics software package, version 17.0 (SPSS Inc., Chicago, IL, USA) and the Stata statistical software package, version 12.0 (StataCorp LP, College Station, TX, USA).

## RESULTS

**Figure 1:**
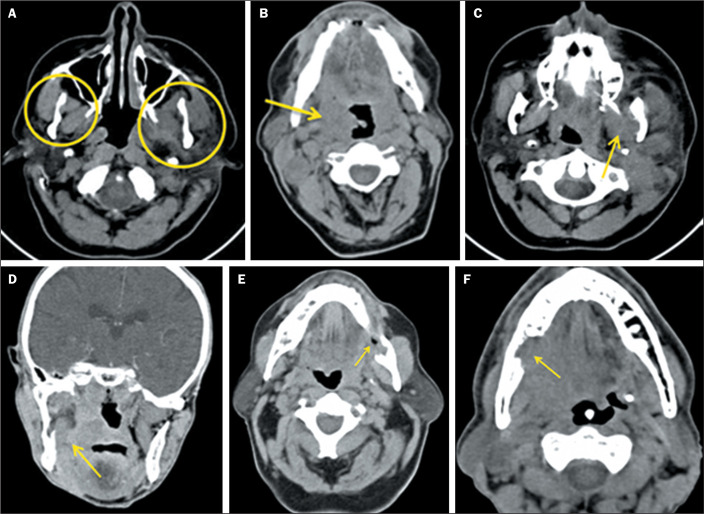
A-C: Axial CT slices with soft-tissue window settings, demonstrating asymmetry in relation to the contralateral masticator space (A), obliteration of the pharyngeal fat plane (B), edema of the medial pterygoid muscle, and invasion of the mandibular canal by the tumor (C). D: Coronal CT slice with a soft-tissue window setting, showing edema of the medial pterygoid muscle. E,F: Axial CT slices with bone-tissue window settings, showing destruction of the mandibular ramus.

The final sample comprised 65 patients, with a predominance of men (90.8%). The mean age was 54.1 ± 7.3 years (range, 39-73 years), and the mean disease duration was 4.9 ± 3.0 months. Examples of the anatomical alterations in the masticator space, as observed on CT, are presented in Figure 1.

The kappa values for interobserver agreement on the CT findings evaluated are shown in Table 1. As can be seen in Table 2, only edema/atrophy of the pterygoid muscles showed a significant association with the presence of trismus (*p* = 0.022). Edema/atrophy of the pterygoid muscles was observed in 60.0% of the patients with trismus, compared with only 21.8% of those without. The risk of developing trismus was 5.4 times greater among the patients who presented edema/atrophy of the pterygoid muscles than among those who did not (Table 2).

**Table 1:** Reproducibility of the CT evaluation.

Finding	Κ	*P*	Interobserver agreement
Asymmetry in relation to the contralateral masticator space	0.146	0.224	Weak
Obliteration of the fat plane	0.457	0.003	Moderate
Edema/atrophy of the pterygoid muscles	0.279	0.024	Fair
Destruction of the mandibular ramus	0.918	< 0.001	Almost perfect

**Table 2:** Distribution of the patients, according to CT findings and the presence of trismus.

Finding	Trismus		Total n (%)	Relative risk	Odds ratio (95% CI)	*P* ^*^
	Yes n (%)	No n (%)				
Asymmetry in relation to the contralateral masticator space Yes	10 (100.0) 5 (50.0)	55 (100.0) 12 (21.8)	65 (100.0) 17 (26.2)	0.42	3.58 (0.89-14.46)	0.111
No	5 (50,0)	43 (78.2)	48 (73.8)	0.12	1.00	
Obliteration of the fat plane Yes	10 (100.0) 8 (80.0)	55 (100.0) 37 (67.3)	65 (100.0) 45 (69.2)	0.22	1.95 (0.37-10.12)	0.711
No	2 (20.0)	18 (32.7)	20 (30.8)	0.11	1.00	
Edema/atrophy of the pterygoid muscles Yes	10 (100.0) 6 (60.0)	55 (100.0) 12 (21.8)	65 (100.0) 18 (27.7)	0.50	5.38 (1.30-22.19)	0.022^†^
No	4 (40.0)	43 (78.2)	47 (72.3)	0.09	1.00	
Destruction of the mandíbular ramus Yes	10 (100.0) 4 (40.0)	55 (100.0) 9 (16.4)	65 (100.0) 13 (20.0)	0.44	3.41 (0.80-14.57)	0.103
No	6 (60.0)	46 (83.6)	52 (80.0)	0.13	1.00	

* Fisher’s exact test.

† Statistically significant.

When we stratified the patients by tumor stage, we found that only the advanced stage (T4) was associated with the presence of trismus (*p* = 0.012). Of the patients with trismus, 90.0% had a T4 tumor, compared with only 43.8% of those without trismus. Patients in stage T4 were found to be 11.6 times more likely to develop trismus than were those in stages T0 to T3 (Table 3).

**Table 3:** Association between tumor stage and trismus.

Stage	Trismus		Total n (%)	Risk	Odds ratio (95% CI)	*P* ^*^
	No n (%)	Yes n (%)				
Any	48 (100.0)	10 (100.0)	58 (100.0)			
T0-T3	27 (56.3)	1 (10.0)	28 (48.3)	0.04	1.00	0.012^†^
T4	21 (43.8)	9 (90.0)	30 (51.7)	0.43	11.57 (1.36-98.67)	

* Fisher’s exact test.

† Statistically significant.

## DISCUSSION

The masticator space is a natural space for the propagation of head and neck neoplasms, particularly those originating in the tonsillar or retromolar region, and to a lesser extent those originating in the nasopharynx or maxillary sinus^**(^10^-^13^)**^. Neoplasms originating in the tonsillar fossa, retromolar trigone, or maxillary sinus affect the masticator space by direct invasion, whereas those originating in the nasopharyngeal region propagate using the natural pathway of the pterygopalatine fossa to access the masticator space^**(^5^)**^.

Trismus is considered a sign of locoregional spread of some head and neck tumors. In a study of patients with malignant head and neck tumors, Ichimura et al.^**(^4^)**^ found that 9% had trismus at the time of diagnosis. Tumors originating in the retromolar trigone can initially infiltrate the tonsillar pillar and spread to the medial pterygoid muscle, causing trismus. In our study, we observed that sign in 15.3% of the patients, and neoplasms of the tonsillar fossa and retromolar trigone collectively accounted for 95% of the neoplasms evaluated. Those primary sites classically present a high prevalence among head and neck tumors, as well as being associated with a higher risk of trismus^**(^6^)**^. Another mechanism described is trismus due to reflex spasm of the muscles, due to infiltration of the mandibular nerve at the level of the foramen ovale^**(^13^)**^.

There is no consensus regarding the criteria for diagnosing trismus^**(^3^,^6^)**^. For Ichimura et al.^**(^4^)**^, the degree of mouth opening must be evaluated by objective measurement, although the cutoff values for that variable in the literature are not uniform, ranging from less than 15 mm to less than 40 mm^**(^14^-^16^)**^. In a meta-analysis of patients undergoing head and neck radiotherapy, Dijkstra et al.^**(^6^)**^ stated that for an accurate assessment of trismus, mouth opening must be measured, because clinical observation alone has low accuracy. However, the authors commented that patient information is relevant in the assessment of functional limitations. Therefore, in the present study, we defined trismus on the basis of the patient complaint and inspection by a medical professional during the physical examination.

In our study sample, 90.0% of the patients with trismus had a tumor staged as T4, whereas only 43.8% of those without trismus had a T4 tumor, and the risk of trismus was 11.6 times greater for T4 staging than for T0-T3 staging. These results support the idea that trismus is related to the locoregional dissemination of tumors, which, in turn, is related to the expansion in depth and laterality of the primary lesion, particularly in neoplasms of the tonsillar fossa and retromolar trigone. Tumors originating in the retromolar trigone do not need to be very large to infiltrate the oropharynx^**(^17^)**^.

There were only three cases of nasopharyngeal neoplasia in our sample, and none of the affected patients presented with trismus. The small number of patients with nasopharyngeal neoplasia precludes any comparisons with the literature. The reported frequency of trismus at the time of diagnosis of such neoplasms is quite variable, ranging from 0% to 36%^**(^7^)**^. Those numbers justified the inclusion of this anatomical site in our study. The nasopharynx does not border the masticator space but communicates with it through the parapharyngeal space, which establishes a natural pathway of anterolateral propagation toward the masticator space^**(^18^)**^. However, the rarity of nasopharyngeal neoplasia in Western populations and the fact that neoplasms originating in the nasopharynx typically spread to other contiguous anatomical regions, such as the base of the skull, the retropharyngeal space, and the prevertebral space, explain the low frequency of masticator space involvement, as reported by Hoe^**(^19^)**^, who found that nasopharyngeal tumors propagated to the masticator space in only 14% of cases.

In our study sample, we did not find any patients with tumors originating in the maxillary sinus. The propagation routes for such tumors are dependent on the epicenter and extent of the lesion, involvement of the masticator space being common. Because maxillary sinus tumors do not typically emit early signs and are therefore usually detected late, extension to neighboring anatomical areas are common. Souza et al.^**(^20^)**^ found extension to the masticator space in 80% of patients with maxillary sinus carcinoma.

A CT scan allows the precise analysis of bone structures, whereas an MRI scan can help differentiate between the tumor itself and inflammatory parts of the tumor mass, and that is important in order to avoid overestimation of the size of the tumor^**(^21^)**^. The use of CT facilitates the assessment of the extent of the tumor extension, the degree of invasion of neighboring anatomical structures, and, notably, the involvement of bone structures by the tumor^**(^20^)**^. For the staging of tumors originating in the tonsillar fossa and evaluating their locoregional extension to soft tissues and bones, which is relevant for the delimitation of malignant lesions in the masticator space, CT is also an excellent method^**(^22^)**^. Galli et al.^**(^1^)**^, comparing CT with MRI, observed that CT had a sensitivity of 100%, compared with 80% for MRI, for detecting primary masticator space lesions, whereas, for the analysis of secondary involvement of the masticator space, CT showed a sensitivity of 90% compared with 100% for MRI.

The assessment of interobserver agreement is important for determining the reliability and reproducibility of CT. The fact that the examiners in the present study had received training in head and neck radiology but did not have extensive experience might have influenced the results. Many studies do not mention the number of examiners involved, and some have only one evaluator^**(^8^,^20^,^23^)**^. However, in most studies that involve more than two examiners, differences of opinion about the images evaluated are resolved by consensus^**(^23^-^27^)**^. In only one of the studies reviewed^**(^28^)**^, the assessment was carried out by more than one examiner and the kappa statistic was calculated in order to estimate the strength of the interobserver agreement. In our study, we found almost perfect agreement between the examiners regarding destruction of the mandibular ramus assessed on CT (κ = 0.918; *p* < 0.001), similar to the results reported in other studies in the literature^**(^23^,^24^)**^.

Cheung et al.^**(^29^)**^ showed that lesions in the masticator space present a typical characteristic on CT, that of anterior to posterior displacement of parapharyngeal fat. In the present study, we found moderate interobserver agreement for obliteration of the fat plane (κ = 0.457; *p* = 0.003). According to Rumboldt et al.^**(^30^)**^, obliteration on CT defines invasion of the masticator space by tumors from the retromolar region. Pascoal et al.^**(^31^)**^ observed lateral displacement, loss of sharpness and even the complete disappearance of fat in tumors of the tonsillar fossa.

In our study, there was weak interobserver agreement for the CT finding of symmetry between the left and right masticator space (Table 1), because there was a lack of agreement between the two examiners (κ = 0.146; *p* = 0.224). The symmetry of the masticator space region, which is composed mostly of soft tissue, is difficult to evaluate, because of the overlapping of structures and subjectivity in the comparison with the normal (contralateral) side. Variations in the positioning of the head of the patient during image acquisition must be considered when evaluating this variable.

Of the patients with trismus in our sample, 60% presented edema/atrophy of the pterygoid muscles on CT, compared with only 21.8% of those without trismus (*p* = 0.022). Of the four variables evaluated, that was the only one that showed a statistical correlation, although the interobserver agreement for edema/atrophy of the pterygoid muscles was weak (κ = 0.279; *p* = 0.024). Ichimura et al.^**(^4^)**^ reported that most patients with tumors of the oral cavity or oropharynx who developed trismus did not show signs of masticatory muscle invasion on CT. According to those authors, such cases of trismus could result from reflex muscle spasm or from CT-undetectable tumor microinvasion into the musculature. That hypothesis might explain why, although 100% of our patients with trismus received a clinical staging of T3 or T4, edema/atrophy of the pterygoid muscles was not seen on CT in all of those patients.

In the present study, we found that trismus was associated only with a stage T4 tumor (*p* = 0.012) and with a CT finding of edema/atrophy of the pterygoid muscles (*p* = 0.022). Predictive calculation showed that the patients with edema/atrophy of the pterygoid muscles had a 5.4 times greater risk of developing trismus than did those without, which underscores the association between this imaging finding and trismus.

In our study sample, trismus showed no statistically significant association with obliteration of the fat plane (*p* = 0.711), asymmetry in relation to the contralateral masticator space (*p* = 0.111), or destruction of the mandibular ramus (*p* = 0.103). Pascoal et al.^**(^31^)**^ found a correlation between the presence of trismus and bone destruction in patients with tumors originating from the tonsillar fossa. Som et al.^**(^26^)**^ observed, using CT, destruction of the mandibular ramus in 80% of patients with neoplasms affecting the masticator space. Some factors may have contributed to our findings: the small number of patients with trismus (n = 10), the type of equipment used, the slice thickness, and the level of experience of the evaluators. Although 3- to 5-mm slices have typically been used in previous studies^**(^26^,^32^)**^, the advantages of multislice CT over single-slice CT include the reduced examination time and the smaller thickness of the slices, which can be as thin as 0.5 mm, thus generating high-definition multiplanar reconstructions. In addition, multislice CT allows the tube voltage and current to be changed, which can result in fewer metallic artifacts, producing images with better definition. Weber et al.^**(^33^)**^ commented that insufficient distribution of the contrast medium among the tumor, muscle and mucous membranes can impair the evaluation of the images, as can metallic artifacts. Linz et al.^**(^34^)**^ compared positron-emission tomography/CT with MRI and with CT alone. The authors found that positron emission tomography/CT showed higher sensitivity, specificity, positive predictive value, and negative predictive value than did the two other modalities, making it an important diagnostic tool in the preoperative staging of squamous cell carcinoma of the oral cavity. According to Maraghelli et al.^**(^35^)**^, multislice CT and MRI are fundamental and complementary in the study and observation of oral infiltration by cavitary diseases, whereas ultrasound and cone-beam CT still play only a marginal role. They also concluded that although positron-emission tomography with fluorine-18-fluorodeoxyglucose does not allow a morphological evaluation like multislice CT and MRI, it is useful in detecting oral neoplasms that are undetectable on conventional imaging, lymph node metastases, other distant metastases, and post-radiotherapy recurrence.

## CONCLUSION

On the basis of our study sample and the methodology employed, we can conclude that obliteration of the fat plane is the most common finding in patients with anatomical alterations in the masticator space due to malignant head and neck neoplasms, followed by asymmetry in relation to the contralateral masticator space and edema/atrophy of the pterygoid muscles. Bone destruction was observed in 20% of our patients. Trismus was present in most of the patients who had a T4 tumor and edema/atrophy of the pterygoid muscles. There is a need for further studies, involving novel methodologies, in order to expand the knowledge in this area.
